# Genotypic variation in transpiration of coppiced poplar during the third rotation of a short‐rotation bio‐energy culture

**DOI:** 10.1111/gcbb.12526

**Published:** 2018-06-04

**Authors:** Alejandra Navarro, Miguel Portillo‐Estrada, Nicola Arriga, Stefan P. P. Vanbeveren, Reinhart Ceulemans

**Affiliations:** ^1^ Research Center of Excellence on Plant and Ecosystems (PLECO) Department of Biology University of Antwerp Wilrijk Belgium; ^2^ Council for Agricultural Research and Economics (CREA), Research Centre for Vegetable and Ornamental Crops Pontecagnano SA Italy

**Keywords:** bio‐energy, leaf hydraulic conductance, *Populus*, stem heat balance method, water potential, water relations, water use efficiency

## Abstract

The productivity of short‐rotation coppice (SRC) plantations with poplar (*Populus* spp.) strongly depends on soil water availability, which limits the future development of its cultivation, and makes the study of the transpirational water loss particularly timely under the ongoing climate change (more frequent drought and floods). This study assesses the transpiration at different scales (leaf, tree and stand) of four poplar genotypes belonging to different species and from a different genetic background grown under an SRC regime. Measurements were performed for an entire growing season during the third year of the third rotation in a commercial scale multigenotype SRC plantation in Flanders (Belgium). Measurements at leaf level were performed on specific days with a contrasted evaporative demand, temperature and incoming shortwave radiation and included stomatal conductance, stem and leaf water potential. Leaf transpiration and leaf hydraulic conductance were obtained from these measurements. To determine the transpiration at the tree level, single‐stem sap flow using the stem heat balance (SHB) method and daily stem diameter variations were measured during the entire growing season. Sap flow‐based canopy transpiration (*E*_c_), seasonal dry biomass yield, and water use efficiency (WUE; g aboveground dry matter/kg water transpired) of the four poplar genotypes were also calculated. The genotypes had contrasting physiological responses to environmental drivers and to soil conditions. Sap flow was tightly linked to the phenological stage of the trees and to the environmental variables (photosynthetically active radiation and vapor pressure deficit). The total *E*_c_ for the 2016 growing season was of 334, 350, 483 and 618 mm for the four poplar genotypes, Bakan, Koster, Oudenberg and Grimminge, respectively. The differences in physiological traits and in transpiration of the four genotypes resulted in different responses of WUE.

## 1 INTRODUCTION

Short‐rotation coppice (SRC) cultures of dedicated woody crops are being developed worldwide for the production of biomass as a renewable bio‐energy source. Among the dedicated woody species for SRC biomass production, poplar (*Populus* spp.) is the most commonly cultivated genus under temperate climate conditions (Stanturf & Van Oosten, 2014). Poplar is, however, sensitive to water deficits and its productivity is closely determined by soil water availability (Marron et al., 2008). Projections of climate change predict an increase in the intensity and the frequency of extreme events (EASAC, 2013). Warmer winters (e.g., 2016) and drier summers (e.g., 2003, 2013) already occurred in Central and Western Europe. Future selection criteria used for SRC poplar genotypes need to take a limited loss of biomass production under water stress conditions into account in combination with minimum water loss by transpiration and a high water use efficiency (WUE—amount of biomass produced per unit of water transpired). Climate change could potentially result in a decrease in biomass production of SRC cultures. Using genotypes with the appropriate physiological features, as for instance effective regulation of stomatal conductance against warming, or high and continuous transpiration rates against flooding, this loss of productivity could be mitigated. These physiological controls must be taken into account, especially as poplar is a fast‐growing species with a broad stomatal response to soil water availability (Pita et al., 2013) and atmospheric humidity (Arango‐Velez, Zwiazek, Thomas, & Tyree, 2011; Silim, Nash, Reynard, White, & Schroeder, 2009). Various physiological traits have been used to investigate the adaptations of poplars to water stress as regulation of leaf stomatal conductance, osmotic adjustments, capacity to repair xylem cavitation, increased biomass allocation to roots, etc. In woody species, xylem vulnerability to cavitation has been extensively used to assess tolerance to drought (Cochard, Casella, & Mencuccini, 2007). Stomatal regulation is a useful mechanism to reduce water loss through transpiration and to optimize water resources when evaporative demand is high (Navarro, Facciotto, Campi, & Mastrorilli, 2014).

Poplar SRC cultivation on marginal lands—drylands and flood plains—can avoid competition with food and feed crops (Gopalkrishnan, Negri, & Snyder, 2011), but the increasing effects of climate change should be taken into consideration. For the cultivation of poplar SRC on lands where soil water availability is subjected to seasonal changes with shortage and excess of water supply, the identification of poplar genotypes with the appropriate physiological traits, WUE and transpiration is important. For most broad‐leaved species transpiration is the major component of water use (Meiresonne, Nadezhdin, Cermak, Van Slycken, & Ceulemans, 1999), and the study of transpirational water loss at all relevant scales (leaf, tree and stand) is thus fundamental. Under natural conditions, the sap flow technique is the only approach for measuring tree transpiration. Different sap flow methods and techniques have been used to measure tree and stand transpiration of poplar, including the stem heat balance (SHB) method (Allen, Hall, & Rosier, 1999; Bloemen et al., 2017; Hall & Allen, 1997; Hall, Allen, Rosier, & Hopkins, 1998; Tricker et al., 2009; Zhang, Simmonds, Morison, & Payne, 1997), the trunk tissue heat balance (THB) method (Hinckley et al., 1994; Petzold, Schwarzel, & Feger, 2011), the heat field deformation (HFM) method (Meiresonne et al., 1999) and the thermal dissipation (TD) method (Kim, Oren, & Hinckley, 2008; Lambs & Muller, 2002; Schmidt‐Walter, Richter, Herbst, Schuldt, & Lamersdorf, 2014; Xi, Di, Wang, Duan, & Jia, 2017). These studies related to poplar stands of longer rotations (Kim et al., 2008; Meiresonne et al., 1999; Petzold et al., 2011; Xi et al., 2017; Zhang et al., 1997), to uncoppiced, single‐stemmed SRC poplar (Bloemen et al., 2017; Hinckley et al., 1994; Schmidt‐Walter et al., 2014; Tricker et al., 2009) as well as to coppiced, multistemmed (Allen et al., 1999; Hall et al., 1998; Tricker et al., 2009) poplars. In all the afore mentioned studies, in particular in the ones on multistemmed SRC poplars, the recorded sap flow measurements, used to obtain stand transpiration, were carried out over a short time period (maximum of eight (Tricker et al., 2009) or 9 weeks (Hall et al., 1998)). No direct, nonmodeled data are available on sap flow measurements, in multistemmed poplar SRC plantation, for an entire growing season. With respect to the above mentioned studies and other related studies that already assessed transpiration in other tree species (Cienciala, Kučera, & Malmer, 2000; Ghimire, Lubczynski, Bruijnzeel, & Chavarro‐Rincon, 2014; Hardanto, Röll, & Hölscher, 2017; Linderson, Iritz, & Lindroth, 2007; McJannet, Fitch, Disher, & Wallace, 2007; O'Brien, Oberbauer, & Clark, 2004), this study determines transpiration at all scales in a long‐term monitored multigenotype and multistemmed poplar SRC plantation.

The temperate European zone is characterized by different and constantly changing climate regimes. So, the future development of SRC poplar plantations requires genotypes with specific adaptive strategies, incl. adaptive potential to cope with the seasonal changes of water supply provoked by the ongoing climatic changes. To achieve this goal, this study intends to assess the genotypic variation in the physiological traits and transpiration of four fast‐growing poplar genotypes, in response to environmental variables in a coppiced SRC plantation in Flanders (Belgium). The main objective is to identify their water use response for future implications of their cultivation under the ongoing climate changes.

## 2 MATERIALS AND METHODS

### 2.1 Study site, plant materials and measurement protocols

**Figure 1:**
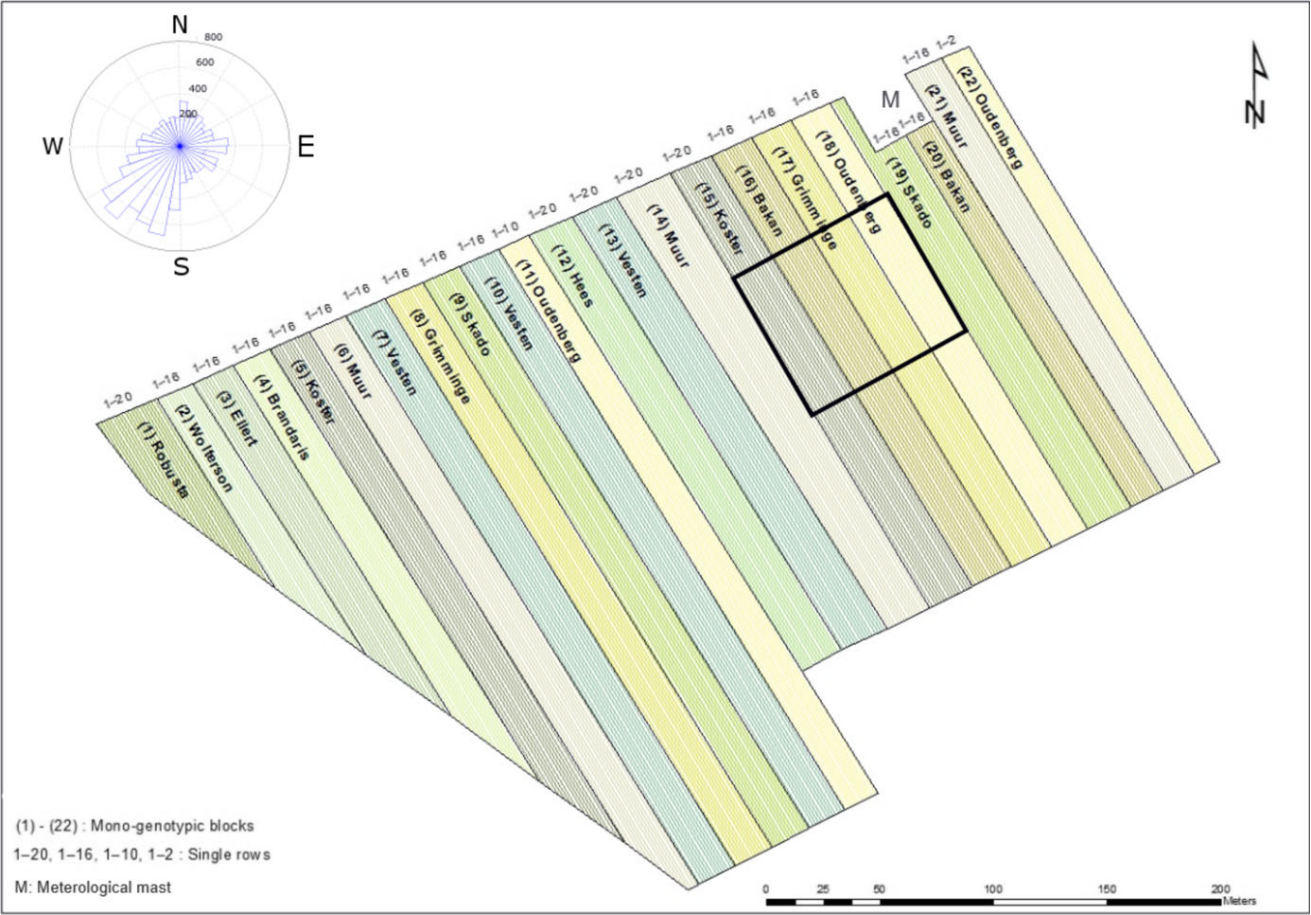
Map of the study site, indicating the location of the meteorological mast (M) on the northeast, as well as the location where trees were equipped with sap flow and dendrometer sensors (black square). In the upper‐left, the wind rose for the period April–December 2016 is shown. The differently colored bands indicate mono‐genotypic poplar blocks in the plantation

Measurements were made in an existing commercial‐scale SRC plantation, established in Lochristi, province East‐Flanders, Belgium (51°06′44″N, 3°51′02″E) at an elevation of 6.25 m above sea level (http://uahost.uantwerpen.be/popfull). Long‐term average (1981–2010) annual temperature at the site is 10°C and the average annual precipitation is 800 mm, evenly distributed over the year (Journée, 2014; Journée, Delvaux, & Bertrand, 2015). The soil has a loamy sand texture (clay content of 11% between 30–60 cm depth) with deeper clay‐enriched sand layers (~75 cm), and is classified as an Anthrosol according to the World Reference Base for Soil Resources (Dondeyne, Vanierschot, Langohr, Van Ranst, & Deckers, 2015; Verlinden, Broeckx, Van Den Bulcke, Van Acker, & Ceulemans, 2013). On 7–10 April 2010 large replicated mono‐genotypic blocks were established with cuttings of 12 selected and commercially available poplar (*Populus*) genotypes (see table 2 in Broeckx, Verlinden, & Ceulemans, 2012). Planting density was 8,000 plants per ha and the total planted area was 9 ha (Figure 1). The planting design consisted of double rows with alternating distances of 0.75 and 1.50 m between the rows and 1.1 m within the row. The site was neither fertilized, nor irrigated. The plantation was coppiced in February 2012 as well as in February 2014, and was in the third rotation during this study. More details on the site, the management, and soil characteristics were previously provided (Broeckx et al., 2012; Verlinden et al., 2013).

An extendable meteorological mast was positioned in the northeastern part of the plantation (Figure 1) at the beginning of June 2010 and provided continuous microclimate and environmental data (Zona et al., 2013). The prevailing wind direction was from the southwest (Figure 1) and all measurements were performed within the footprint on the upwind side of the mast. These measurements were confined to a subset of four genotypes (three individual trees per genotype), all close to the mast (<50 m). The selected genotypes were characterized by a different parentage: Bakan (parentage *Populus trichocarpa* T. & G. × *P. maximowiczii* A. Henry), Oudenberg (parentage *P. deltoides* Bartr. ex Marsh. × *P. nigra* L.), Koster (parentage *P. deltoides* Bartr. ex Marsh. × *P. nigra* L.), and Grimminge (parentage *P. deltoides* Bartr. ex Marsh. × (*P. trichocarpa* T. & G. × *P. deltoides* Bartr. ex Marsh.). The four selected genotypes were chosen because of their wide genetic background, their different growth pattern (e.g., number of shoots per stool, shoot diameter), and because they are hybrids of the mostly cultivated poplar species (i.e., *P. trichocarpa*; Euramerican = *P. deltoides* × *P. nigra*; and *P. maximowiczii*). These four hybrid genotypes are of interest to the scientific community as they represent the most planted hybrid poplars. More details on the origin, the family and the gender of these genotypes were previously provided (Broeckx et al., 2012). All measurements were made on multistemmed trees as the plantation had already been coppiced twice, during the entire growing season of 2016 (from April to November), i.e., during the seventh growth year of the plantation and before the third coppice (in February 2017). The average height of the canopy was 6.2 m in the middle of the growing season.

### 2.2 Environmental variables

Environmental variables were continuously recorded at the site: air temperature (*T*_air_; °C) and relative humidity (RH_air_; %) were recorded on the extendable mast at 7.7 m above the ground surface using Vaisala probes (HMP 45C, Vaisala, Helsinki, Finland); these data were used to calculate the vapor pressure deficit (VPD; kPa). Incoming photosynthetically active radiation (PAR; 400–700 nm) was measured at the same height using a quantum sensor (LI‐190, Li‐COR, Lincoln, NE, US). Global radiation (*R*_g_; W/m^2^) was measured with a pyranometer (CM3 component of CNR4 radiometer, Kipp & Zonen, Delft, the Netherlands). Precipitation (mm) was recorded using a tipping bucket rain gauge (3665 R, Spectrum Technologies Inc., Plainfield, IL, USA). Soil water content (SWC; m^3^/m^3^) was measured at five different spatial locations, each one with a vertical profile of five different depths (5, 10, 20, 50 and 100 cm from the soil surface), using time domain reflectometer (TDR) probes (CS616; Campbell Scientific, Logan, UT, USA) inserted horizontally. Values of SWC at depths of 5, 10 and 20 cm were averaged and represented as SWC at 0–20 cm depth. Soil water table depth (WTD; cm below the surface) was measured in the proximity of each SWC profile with a pressure transducer (CS451; Campbell Scientific Inc.) installed in PVC pipes vertically inserted into the ground to a depth of approximately 2 m. All measurements were logged continuously through the combination of a data logger (CR1000; Campbell Scientific Inc.) and multiplexers (AM 16/32B; Campbell Scientific Inc.). For each variable, values were sampled once every minute and averaged every 30 min. If an instrument occasionally failed, the missing environmental variable (*T*_air_, *RH*,* R*_g_, PAR or precipitation) was gap‐filled using data from nearby meteorological stations operated by the Royal Meteorological Institute of Belgium (RMI) at 8 and 10 km from the research site, respectively, in Zelzate (51°10′53″N, 3°48′19″E) and Melle (50°58′51″N, 3°49′03″E).

### 2.3 Leaf level measurements

Water relations and gas exchange were determined every 15–20 days between early May and end September 2016 (i.e., the measurement campaign), resulting in a total of 10 days characterized by different incoming PAR and VPD conditions. The diurnal courses of leaf stomatal conductance to water vapor (*g*_s_; mmol m^−2^ s^−1^) were measured with a steady‐state diffusion porometer (Delta‐T AP4, Delta‐T Devices Ltd, Burwell, Cambridge, UK). Three mature leaves of a similar age were randomly chosen from the upper canopy level (Heilman, Hinckley, Roberts, & Ceulemans, 1996; Hinckley et al., 1994), and measurements were taken at 2–3 hr intervals from sunrise to sunset. Access to the canopy was guaranteed by ladders with a working height of 5.2 m. Leaf transpiration (*E*_leaf_; mg H_2_O m^−2^ s^−1^) was calculated as follows:

(1) $$\begin{array}{cc} & E_{\mathrm{leaf}}=g_{s}\times \Delta C_{\mathrm{wv}\;\mathrm{leaf}-\mathrm{air}}=g_{s}\\ & \times \left[C_{\mathrm{wv}\;\mathrm{sat}.\mathrm{leaf}}-\left(C_{\mathrm{wv}\;\mathrm{sat}.\mathrm{air}}\times \frac{{\text{RH}}_{\mathrm{air}}}{100}\right)\right]\times 1,000 \end{array}$$

where *g*_s_ = leaf stomatal conductance to water vapor (m/s) and Δ*C*_wv leaf‐air_ = concentration difference in water vapor between the intercellular spaces in the leaf and the atmosphere (g/m^3^).

Measurements of midday leaf water potential (Ψ_l_; MPa) and midday stem water potential (Ψ_*x*_; MPa) were performed between 10:00 and 12:00 hours using one developed leaf of similar age and position in the canopy (8–12 leaves from the apex) on three trees per genotype. For the determination of Ψ_*x*_, leaves were covered with both a plastic bag and aluminum foil for at least 2 hr before the measurement. Bagging prevented leaf transpiration, allowing the leaf water potential to equal the stem water potential (Begg & Turner, 1970). The leaves sampled for Ψ_l_ and Ψ_*x*_ were cut and immediately placed in a pressure chamber (ARIMAD‐2; A.R.I. Kfar Charuv‐Water Supply Accessories, Ramat Hagolan, Israel) in the field according to Scholander, Hammel, Bradstreet, and Hemmingsen (1965). The pressure in the pressure chamber was raised using nitrogen gas at a rate of 0.03 MPa/s.

Leaf water transport capacity can be quantified in terms of leaf hydraulic conductance (*K*_leaf_; mg m^−2^ s^−1^ MPa^−1^). This *K*_leaf_ was estimated from the values of *E*_leaf_, Ψ_*x*_ and Ψ_l_ as follows:

(2) $$K_{\mathrm{leaf}}=E_{\mathrm{leaf}}/\Delta \Psi_{x-1},$$

where ΔΨ_*x*–l_ is the difference between midday stem and leaf water potential (MPa).

### 2.4 Tree level measurements: sap flow

Sap flow rates (*F*_s_; g/hr) of individual stems were measured using the stem heat balance (SHB) technique (Baker & van Bavel, 1987; Sakuratani, 1981), as already successfully applied in previous studies on SRC (Allen et al., 1999; Bloemen et al., 2017; Hall et al., 1998; Tricker et al., 2009). *F*_s_ was continuously monitored on two stems per tree and three trees per genotype throughout the entire growing season (9 April to 12 November 2016), using 24 SHB sap flow sensors able to operate in a wide range of stem diameters (SGEX16, SGEX19, SGEX25 and SGB35, Dynamax Inc., Houston, TX, US). Individual stems on the trees were selected to be representative of the entire range of stem diameters measured at 0.22 (*d*) and at 1.30 m height (diameter at breast height—*DBH*) during an extensive inventory performed in February 2016. The sensors were mounted at a height of 1.50 m above the soil surface, always below the first branch of each stem and installed following the manufacturer's instructions (Dynagage sap flow sensors manual, Dynamax Inc., Houston, TX, USA). Additionally, the sensors were thermally insulated from the environment with the weather shields supplied by the manufacturer and several layers of insulating aluminum foil wrapped around the sensor. Finally, conical funnels of appropriate diameters were placed upside down around the stems above each sensor, thus preventing water entering the sensors during rainy periods (for installation see https://youtu.be/s7Zz5aNApLI and https://www.uantwerpen.be/en/rg/pleco/research/research-projects/marie-curie/physio-pop/).

*F*_s_ was calculated from the raw data according to the standard procedure for SHB sensors as previously described in detail (Baker & van Bavel, 1987; Sakuratani, 1981, 1984; Steinberg, van Bavel, & McFarland, 1990). The low‐temperature cut‐off filter and the high flow filter were applied as described in the manufacturer's manual (Dynagage sap flow sensors manual, Dynamax Inc.). As poplars are fast‐growing trees, the increase in stem surface area during the growing season was taken into account in the energy balance equations by including the increase in stem diameter recorded by automatic point dendrometers (ZN11‐O‐WP, Natkon, Hombrechtikon, Switzerland). The dendrometers were installed with a ring‐shaped carbon fiber frame at a height of 1.30 m, just below the sap flow sensors. Data from the sap flow sensors and the dendrometers were collected at 30‐s intervals with a data logger (CR1000; Campbell Scientific, UK) and averaged every 30 min.

**Figure 2:**
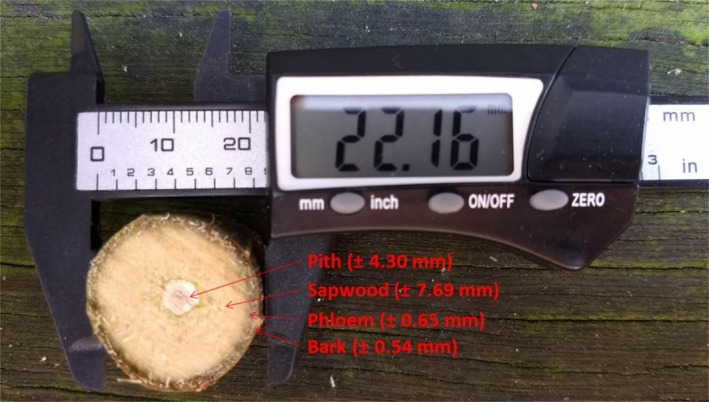
Stem cross‐section of short‐rotation coppice poplar showing the thickness layers of tissues

Stem transpiration (*E*_stem_; g H_2_O m^−2^ s^−1^) was obtained by dividing *F*_s_ (g/hr) by the sapwood area (Figure 2) at 0.22 m above the soil surface (*A*_s_; m^2^) of each stem. *E*_stem_ of each genotype is the final result after averaging six stems per genotype. *A*_s_ was estimated using the *DBH* from the dendrometers and converting them to stem diameters at 0.22 m (*d*; mm; Pontailler, Ceulemans, Guittet, & Mau, 1997) via allometric equations (see Supporting Information Table S1).

(3) $$E_{\mathrm{stem}}=\frac{F_{s}}{A_{s}}\times \frac{1}{3,600}$$

to analyze the relationship of *F*_s_ with VPD and PAR, *F*_s_ was cumulated daily (kg/day) and expressed relatively to the daytime‐averaged VPD (with daytime defined as periods when PAR > 5 μmol m^−2^ s^−1^) or to the daily cumulated PAR (mol m^−2^ s^−1^). The relationship between *F*_s_ and VPD was analyzed according to Tang et al. (2006) and Ewers, Oren, Johnsen, and Landsberg (2001) by fitting the following exponential saturation equation:

(4) $$F_{s}=a\times \left(1-e^{-b\times \mathrm{VPD}}\right),$$

where *a* (kg/day) and *b* (k/Pa) correspond to the fitted coefficients, *F*_s_ is approaching to *a* at a very high VPD. A linear regression was used to analyze the relationship between *F*_s_ and PAR.

### 2.5 Stand level measurements

To obtain the canopy transpiration and, therefore, the daily transpiration rate per unit of ground area (*E*_c_; mm/day) of each genotype, the transpiration of the entire tree was quantified. This whole‐tree transpiration was estimated by summing the *F*_s_ contributions from all stems. The multiple stems of each tree of each genotype were classified in terms of the operative stem diameter of every sap flow sensor (see Supporting Information Table S1) after a detailed inventory of the number of stems per stool and stem diameters made in January 2016, that is, during the dormant stage (see Supporting Information Figure S1 for further details). The sap flow rate per unit of sapwood area (*F*_*ij*_; g m^−2^ hr^−1^) for the *i*th stem of the *j*th range was obtained by dividing *F*_s*ij*_ (g/hr) by the sapwood area (Figure 2) at 0.22 m above the soil surface (*A*_s*ij*_; m^2^). The sum of all *F*_ij_ was multiplied by the ratio of the average sapwood area at 0.22 m (*A*_s‐avg_; m^2^) for all stems equipped with dendrometers to the ground surface area per tree (*SA*; m^2^), and finally the result was transformed to kg m^−2^ h^−1^ and therefore approximated to mm of water column per hour (mm/hr), as follows:

(5) $$E_{c}=\left(\sum_{j=1}^{4}\sum_{i=1}^{n}\left(\frac{F_{\mathrm{sij}}}{A_{\mathrm{sij}}}\right)\times \frac{A_{s-\mathrm{avg}}}{\mathrm{SA}}\right)\times 10^{-3}\text{kg/g},$$

where *j *= operative stem diameter range, *i *= stem number and *n *= the number of stems per operative stem diameter range. Further on, all the hourly values of *E*_c_ (mm/hr) of each day were summed to obtain the daily transpiration rate per ground area (*E*_c_; mm/day).

Both *A*_s_ and *A*_s‐avg_ were estimated using the stem *DBH* from the dendrometers and converting them to stem *d* via allometric equations. The *SA* was estimated for each genotype based on the tree density and on the mortality of trees in each mono‐genotypic poplar block of the site (see Supporting Information Table S1 for further details).

To obtain *F*_s_ and *E*_c_ the SHB technique provided data of high time resolution with rather few gaps. Notwithstanding this positive aspect, the long recording period in the present study (6 months) involved a number of practical problems: (a) the SHB sap flow sensors had to be changed throughout the growing season from a smaller to a larger sensor, as the poplars grew very fast, and (b) the occurrence of adventitious roots and mold on the stem section where the sensor was positioned, implied the occasional removal of the sensors for cleaning both, and the re‐assembly afterward.

Water use efficiency (WUE; g aboveground dry matter per kg water transpired) was calculated for each poplar genotype from the quotient of the dry biomass yield (DB) and *E*_c_. DB was estimated in terms of aboveground woody biomass (AGWB) for each mono‐genotypic block after a detailed inventory of stems and stem diameters. The number of stems per stool was counted for every stool in one row per mono‐genotypic block, and the stem *d* was measured for all stems of every fifth stool in the same row. Stem *d* was measured with a digital calliper (Mitutoyo, CD‐15DC, UK; accuracy of 0.01 mm). The inventory was made in January 2017, that is, during the dormant stage. From these data, the stool mortality at the time of coppicing (in February 2017) was calculated and a genotype‐specific allometric relation was established between *d* and AGWB (Verlinden, Broeckx, & Ceulemans, 2015). This allometric relation was established by manually harvesting 10 random stems per genotype, covering the entire stem diameter range. Stems were cut at 7 cm height (i.e., to approximate the expected harvesting height) and weighed with a precision scale (Kern MH10K10, Kern & Sohn GmbH, Germany; accuracy of 1 g). Their exact *d* was also determined with a digital calliper, and a power function was fitted to the data to obtain the AGWB per stem as a function of *d*, from the equation:

(6) $$\text{AGWB}=a\times d^{b}$$

the AGWB per stool was obtained by summing the AGWB of all stems on each of the sampled stools. The inventory data were considered spatially representative per mono‐genotypic block and therefore an average AGWB per stool was quantified. Taking the mortality into account, this value was multiplied with the surviving density per mono‐genotypic block to obtain the actual DB.

### 2.6 Statistical analyses

For each measurement day, differences in *E*_leaf_ among genotypes were tested separately hour by hour in a one‐way ANOVA. When the differences were significant among genotypes (*p *≤* *0.05), Tukey's Honestly Significant Difference (HSD) test was used to do pairwise comparisons. This statistical analysis was performed using the statistical software package statgraphics plus 5.1 (StatPoint Technologies Inc., Warrenton, VA, USA).

Data of *E*_stem_ were similarly tested with one‐way repeated measures ANOVA on Ranks test at a significance level of *p *<* *0.001. A subsequent Tukey HSD's pairwise comparison (*p *≤* *0.05) was used to isolate different groups. The statistical tests, curve fittings and regression analyses were performed using the statistical software sigmaplot 13.0 (Systat Inc., San Jose, CA, USA).

## 3 RESULTS

### 3.1 Genotypic differences in physiological responses

**Figure 3:**
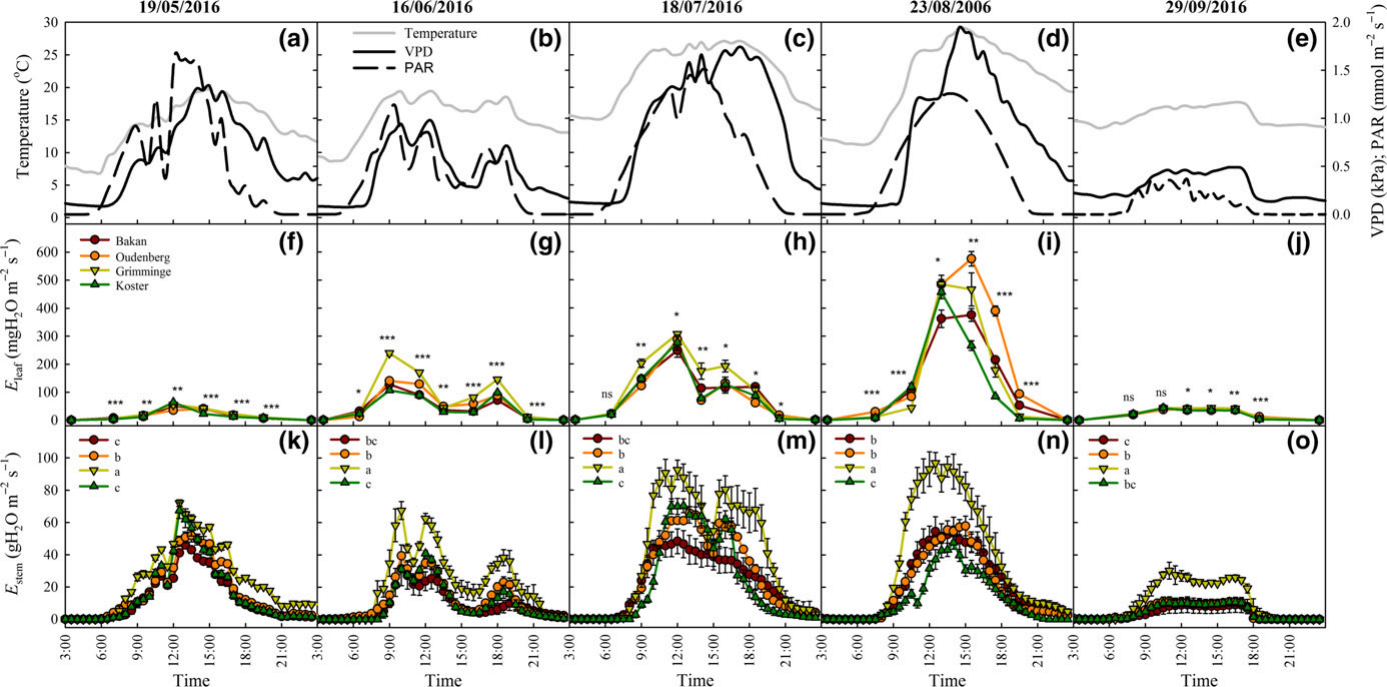
Diurnal courses of meteorological variables, of leaf transpiration (*E*_leaf_) and of stem transpiration (*E*_stem_) for five specific days of the 2016 growing season for poplar genotypes Bakan (dark red circle—line), Oudenberg (orange circle—line), Grimminge (light green triangle—line) and Koster (dark green triangle—line). Top panels (a–e): temperature (gray solid line), photosynthetically active radiation (PAR, black dashed line) and vapor pressure deficit (VPD, black solid line). Middle panels (f–j): leaf transpiration (*E*_leaf_). Bottom panels (k–o): stem transpiration (*E*_stem_). *, **, *** denote statistically significant differences at the 0.05, 0.01 and 0.001 levels respectively; ns: no significant differences. Different letters denote significant differences among genotypes according to Tukey HSD's test (*p *≤* *0.05). Vertical bars represent standard errors of the means

The diurnal courses of *E*_leaf_ and *E*_stem_ of the four SRC poplar genotypes covered a wide range of weather conditions typical of the Belgian climate, in terms of temperature, PAR and VPD (according to the Royal Meteorological Institute of Belgium (RMI), https://www.meteo.be; Figure 3). The dynamics of *E*_leaf_ and *E*_stem_ followed the PAR and VPD patterns (Figure 3). This was more evident for *E*_stem_ than for *E*_leaf_ as *E*_stem_ values were recorded every 30 min while *E*_leaf_ was determined every 2–3 hr (Figure 3). The highest values of transpiration (leaf and stem) were reached during the summer months (Figure 3h,i,m,n) coinciding with the maxima of VPD (1.73 and 1.95 kPa on 18 July and 23 August, respectively, Figure 3c,d). The lowest daytime transpiration values were observed on 29 September (Figure 3j,o) as a result of the low values of PAR and VPD on that day (max. PAR values of only 0.368 mmol m^−2^ s^−1^ and a VPD of 0.5 kPa; Figure 3e). The diurnal courses of *E*_leaf_—but especially of *E*_stem_—in Grimminge differed significantly from the other genotypes across the growing season, primarily due to overall higher transpiration values during daytime (Figure 3f–o) and to a sustained transpiration in the late hours of the day after the decline of PAR (Figure 3m,o). Over the entire growing season, genotypes Bakan and Koster had the lowest values of both *E*_leaf_ and *E*_stem_ (Figure 3f–o).

**Figure 4:**
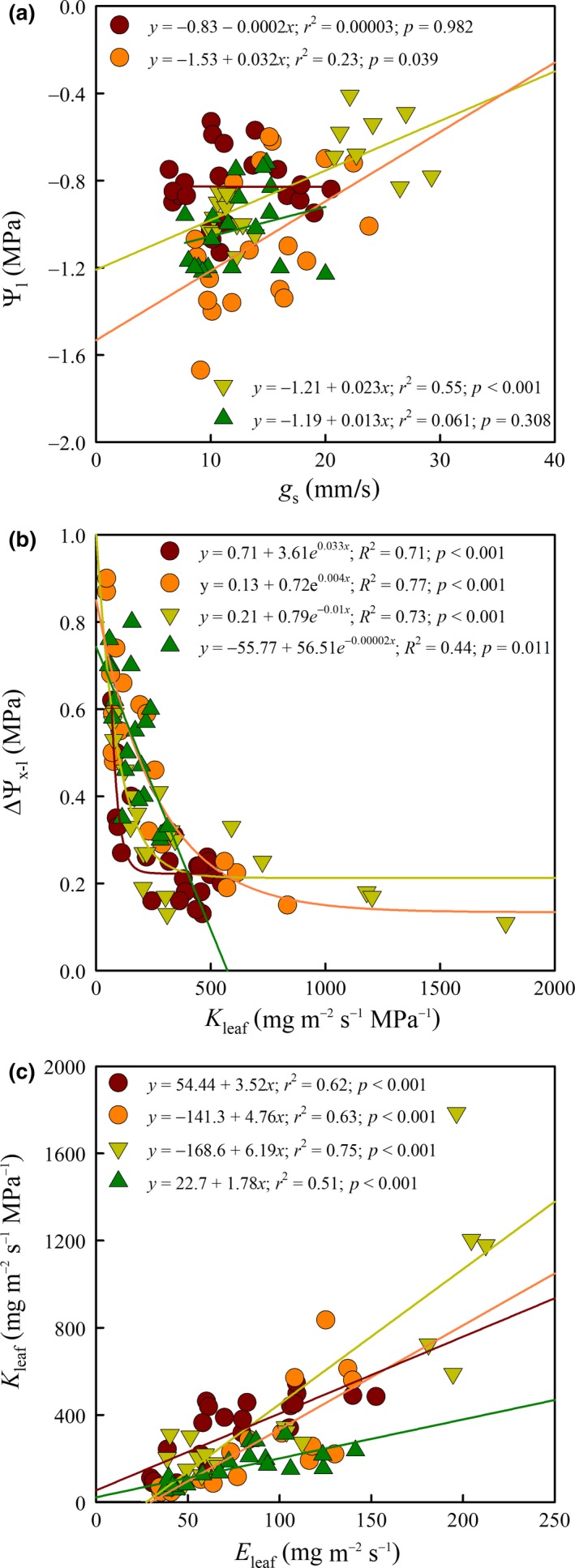
Water relations and leaf hydraulic behavior of poplar genotypes Bakan (dark red circle—line), Oudenberg (orange circle—line), Grimminge (light green triangle—line) and Koster (dark green triangle–line) during the 2016 growing season. Top panel (a): stomatal conductance (*g*_s_) as a function of midday leaf water potential (Ψ_l_). Middle panel (b): leaf hydraulic conductance (*K*_leaf_) as a function of the difference between midday stem and leaf water potential (ΔΨ_*x*–l_). Bottom panel (c): leaf transpiration (*E*_leaf_) as a function of *K*_leaf_. Lines represent linear relationships (*y *= a + b*x*; top and bottom panels) and exponential decay curves (*y *= a + b*e* ^c*x*^; middle panel)

The relation between *g*_s_ and Ψ_l_ (Figure 4a) differed among the four genotypes. An increase in *g*_s_ was associated with an increase in Ψ_l_ in Oudenberg and in Grimminge (Figure 4a), the linear correlation being stronger in Grimminge (*r* ^2^ = 0.55; *p *<* *0.001) and weaker in Oudenberg (*r* ^2^ = 0.23; *p *=* *0.039). In Koster and Bakan there was no correlation between *g*_s_ and Ψ_l_, as Ψ_l_ was not related to the changes in *g*_s_ over the course of the 2016 growing season (Figure 4a). In both genotypes Ψ_l_ changed only very slightly (from −0.7 to −1.2 MPa in Koster, and from −0.5 to −1.1 MPa in Bakan).

There were also significant genotypic differences in the relation of ΔΨ_*x*–l_ as a function of *K*_leaf_ and in the relation of *K*_leaf_ as a function of *E*_leaf_ (Figure 4b,c). The nonlinear regressions for ΔΨ_*x*–l_ – *K*_leaf_ (Figure 4b) and the linear regressions for *K*_leaf_ – *E*_leaf_ (Figure 4c) illustrated how—in genotypes Grimminge and Oudenberg—an increase in *K*_leaf_ was related to a low ΔΨ_*x*–l_, and an increase in *E*_leaf_ related to an increase in *K*_leaf_ (Figure 4b,c). Grimminge had the highest *K*_leaf_ value (1800 mg m^−2^ s^−1^ MPa^−1^) with the lowest ΔΨ_*x*–l_ (0.11 MPa) and the highest *E*_leaf_ (200 mg m^−2^ s^−1^) as compared to the other genotypes (Figure 4b,c).

In Koster and Bakan *K*_leaf_ remained almost unaffected by a low or high ΔΨ_*x*–l_ (Figure 4b), but the nonlinear equations differed between both genotypes (Figure 4b). The Koster *K*_leaf_ dataset had a shorter range (20–300 mg m^−2^ s^−1^ MPa^−1^) compared to the Bakan dataset (50–600 mg m^−2^ s^−1^ MPa^−1^), and a higher *K*_leaf_ was reached in Bakan than in Koster (600 vs. 300 mg m^−2^ s^−1^ MPa^−1^) with lower values of ΔΨ_*x*–l_ (Figure 4b). The relationships between *E*_leaf_ and *K*_leaf_ were very similar for Koster and Bakan, and *K*_leaf_ increased at a low rate with increasing *E*_leaf_ (Figure 4c). In both last mentioned genotypes—but especially in Koster—*K*_leaf_ values (20–300 mg m^−2^ s^−1^ MPa^−1^) were lower compared to Grimminge and Oudenberg throughout the entire growing season. In both cases (Figure 4b,c), the correlation for Koster (*R* ^2^ = 0.44; *p *=* *0.011 and *r* ^2^ = 0.51; *p *<* *0.001, respectively) was always weaker than for Bakan and the two other genotypes.

### 3.2 Genotypic differences in relation to environment

**Figure 5:**
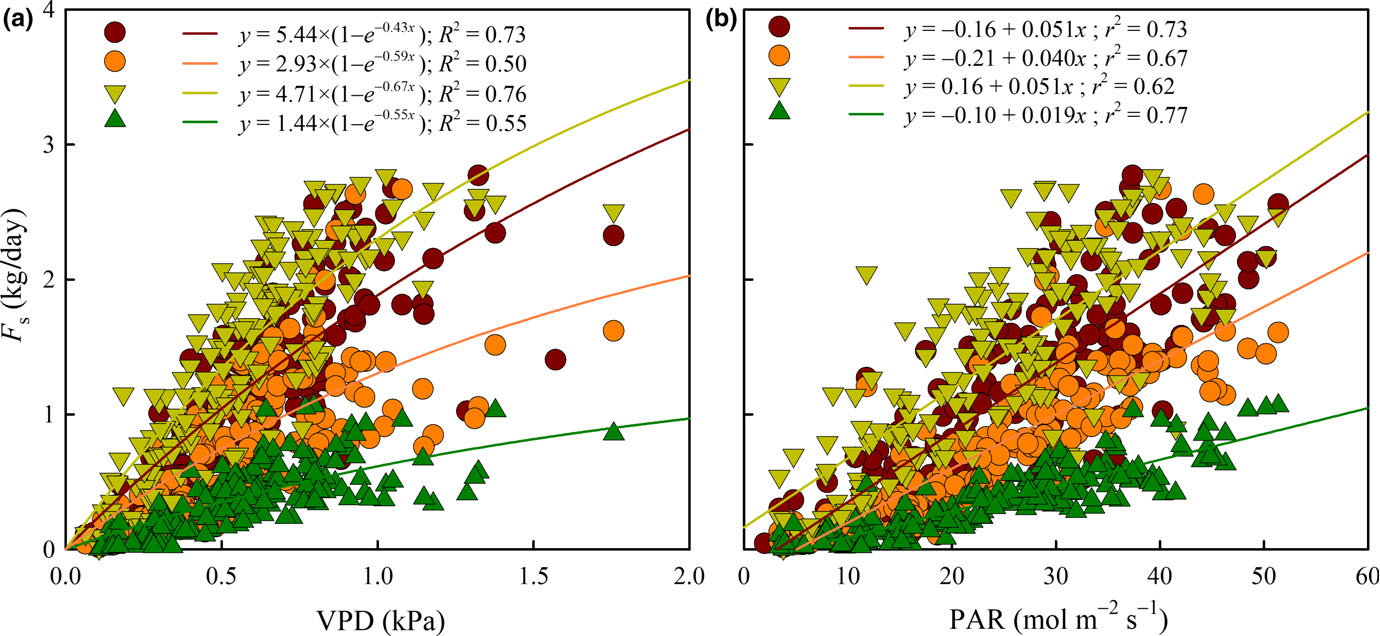
Daily cumulative sap flow rate (*F*_s_) of genotypes Bakan (dark red circle—line), Oudenberg (orange circle—line), Grimminge (light green triangle—line) and Koster (dark green triangle—line), as a function of daytime‐averaged vapor pressure deficit (VPD; left panel, a) and daily cumulative photosynthetically active radiation (PAR; right panel, b). In the left panel, lines are exponential saturation curves and in the right panel, lines represent linear regression lines. All fitting equations were highly significant (*p *<* *0.001)

Daily sums of *F*_s_ were significantly correlated with daytime‐averaged VPD (Figure 5a, p* *<* *0.001). The maximum daily *F*_s_, estimated from coefficient “*a*” for an infinite VPD in Eqn. (4), was higher for Bakan and Grimminge (5.44 and 4.71 kg/day, respectively) than for Oudenberg (2.93 kg/day) and Koster (1.44 kg/day). The strongest correlations between *F*_s_ and VPD (Figure 5a) were observed for Bakan and Grimminge (*R* ^2^ > 0.73). A similar genotypic difference was observed for the *F*_s_ vs. PAR regression (Figure 5b), but in this case, the strongest correlation was observed for Koster and Bakan (*r* ^2^ = 0.77 and 0.73, respectively) and the weakest for Grimminge (*r* ^2^ = 0.62).

### 3.3 Canopy transpiration, WUE and biomass productivity

**Figure 6:**
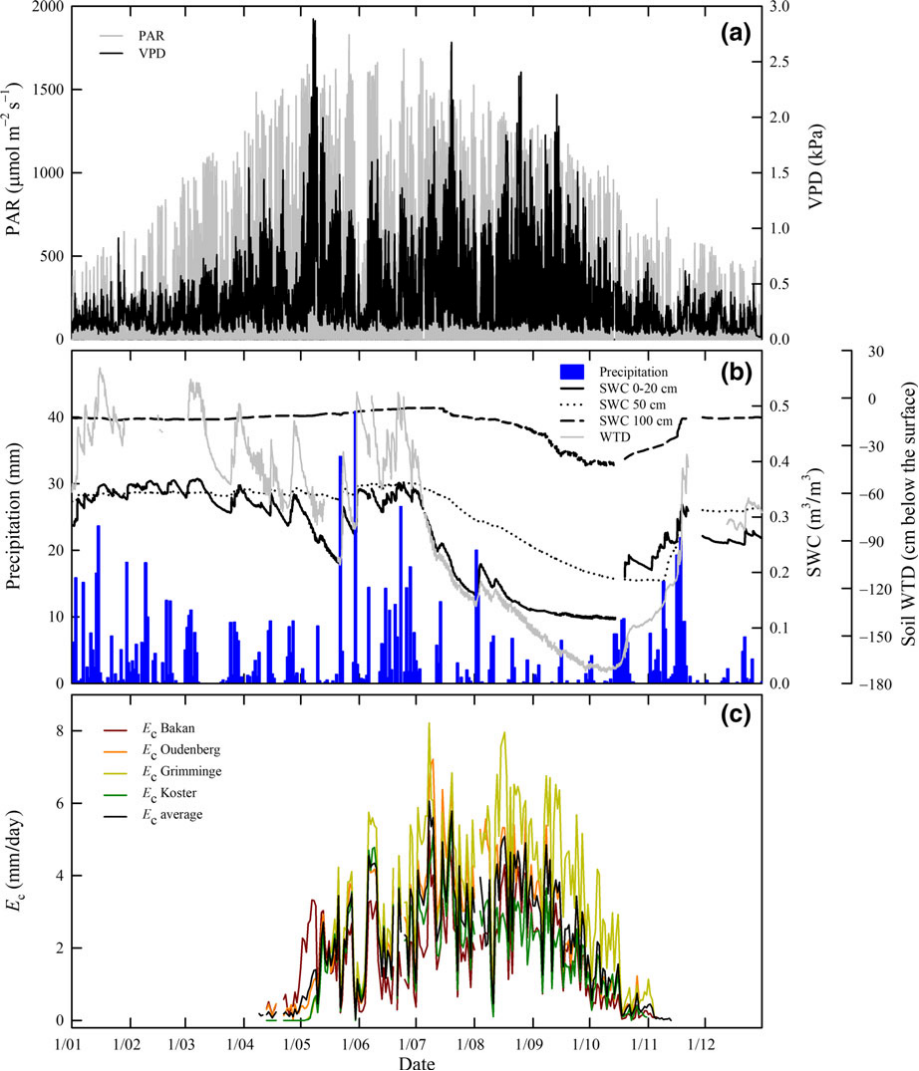
Time course of meteorological variables and of daily sap flow‐based canopy transpiration (*E*_c_) during the year 2016. Top panel (a): photosynthetically active radiation (PAR; gray line) and vapor pressure deficit (VPD; black line). Middle panel (b): daily summed precipitation (blue bars), soil water content (SWC) measured at 0–20 cm (black solid line), 50 cm (black dotted line) and 100 cm (black dashed line) depth and soil water table depth (Soil WTD; gray line). Bottom panel (c): *E*_c_ Bakan (dark red line), *E*_c_ Oudenberg (orange line), *E*_c_ Grimminge (light green line), *E*_c_ Koster (dark green line) and average *E*_c_ of the four genotypes (black line)

The weather conditions during the year 2016 and especially during the growing season were very variable (Figure 6a). VPD and PAR showed very low values (<0.3 kPa and <400 μmol m^−2^ s^−1^, respectively) during the daytime period of the growing season from 30 May till 5 June 2016 and at the beginning of August, with minima of 0.1 kPa for VPD and 30 μmol m^−2^ s^−1^ for PAR on 30 May, concurring with maxima of 40 mm for precipitation during that day (Figure 6a,b). Maximum values of VPD were reached on 7 May (2.9 kPa) and on 19 July (2.7 kPa), and values of 1.5 kPa were exceeded during most of the days between 17 August and 15 September; these VPD values coincided with periods of low precipitation (Figure 6a,b). As a result of the frequent and intense precipitation events along the year, the SWC measured at 0–20 cm depth was always higher than 0.2 m^3^/m^3^, except for 3 months (from end of July till end of October 2016); SWC never reached below 0.1 m^3^/m^3^. The decrease in SWC at 50 cm depth was less pronounced and almost imperceptible for SWC at 100 cm depth (Figure 6b). The soil WTD followed the trend of the SWC measured at 0–20 cm depth, reaching also the minimum values from end of July till end of October 2016 (Figure 6b).

Transpiration was tightly connected to the phenological stage of the trees; the onset of spring (leaf area development) and leaf fall in late autumn were easily identifiable from sap flow measurements with clear differences among the four genotypes. The onset of transpiration took place on 9 April in Bakan and Oudenberg (Figure 6a), *E*_c_ being higher than 1 mm/day in Bakan from 20 April onward and in Oudenberg only 15 days later, on 6 May (Figure 6c). The transpiration of Grimminge and Koster started 1 month later (i.e., on 5 May) with respect to the two aforementioned genotypes, immediately reaching values higher than 1 mm/day (on 11 May). The daily *E*_c_ dynamics for the four poplar genotypes were correlated with the changes in VPD and PAR (Figure 6a,c). The maximum daily *E*_c_ values were reached on 8 July (Figure 6c) for all genotypes, but Grimminge and Oudenberg (8.2 and 7.2 mm/day, respectively) transpired around 3 and 2 mm more than Bakan and Koster (5.2 and 5.6 mm/day, respectively). On average *E*_c_ reached values >5.5 mm/day on 8 and 20 July (Figure 6c), which coincided with the daily maxima of PAR and VPD on these days (Figure 6a) and 4 mm/day during the period between 17 August and 15 September (Figure 6c) concurring with the high VPD and the low precipitation in this period (Figure 6a,b) and with a decrease in the SWC and in the soil WTD (Figure 6b). Bakan and Koster showed the lowest daily transpiration values (avg. = 1.6 and 1.8 mm/day, respectively) while Grimminge transpired twice as much (avg. = 3.1 mm/day), and Oudenberg showed intermediate values of daily transpiration (avg. = 2.5 mm/day).

The total precipitation in 2016 was 946 mm, evenly distributed over the year, although maximum values were reached during the spring, and minima at the end of summer (Figure 6b). This value of precipitation was 115% higher than the total average *E*_c_ during the 2016 growing season (441 mm).

The total DB for the seventh growth year of the plantation at the end of the third rotation was highest for Bakan and Koster, lowest for Grimminge and intermediate for Oudenberg (Table 1). This ranking of genotypes was quite the opposite as for the total *E*_c_ during the 2016 growing season (9 April to 12 November 2016). Indeed, most water was transpired by Grimminge (620 mm), the least by Bakan and Koster (334 and 350 mm), and Oudenberg (483 mm) took an intermediate place in terms of water use (Table 1). Given the lower seasonal *E*_c_ and the higher biomass yield, WUE was higher for Bakan and Koster (7.15 and 6.24 g/kg, respectively) than for Oudenberg (4.13 g/kg), and especially than for Grimminge (only 2.34 g/kg).

**Table 1:** Seasonal dry biomass yield (DB), total sap flow‐based canopy transpiration (*E*_c_) and water use efficiency (WUE; g aboveground dry matter per kg water transpired) of the four poplar genotypes during the 2016 growing season. DB represented the total amount of biomass after 3 years in the third rotation of the plantation

Poplar genotype	DB (Mg/ha)	*E*_c_ (mm)	WUE (g/kg)
Grimminge	14.48	618	2.34
Oudenberg	19.96	483	4.13
Bakan	23.87	334	7.15
Koster	21.84	350	6.24

## 4 DISCUSSION

### 4.1 Genotypic differences at leaf and tree levels

The four genotypes responded differently to the environmental and soil conditions during the growing season, as indicated by their significantly different physiological response. Previous studies indicated that these differences among poplar genotypes are driven by variation in *g*_s_ (Fichot et al., 2011; Monclus et al., 2006; Navarro et al., 2014). The differences in *g*_s_ among genotypes were clearly manifested in the values of *E*_leaf_ and *E*_stem_ observed in this study. The low values of *g*_s_ in Bakan agreed with the low values of *g*_s_ observed at the same site, but in the second year of the plantation (2011; single stem) for Bakan and Skado (*P. trichocarpa *× *P. maximowiczii*; Broeckx, Fichot, Verlinden, & Ceulemans, 2014). Leaves of *P. deltoides* (female parent of Oudenberg, Grimminge and Koster, but also 50% of the male parentage of Grimminge) have more stomata per leaf surface than leaves of *P. trichocarpa* (female parentage of Bakan; Al Afas, Marron, & Ceulemans, 2005; Pearce, Millard, Bray, & Rood, 2005; Dillen, Marron, Koch, & Ceulemans, 2008), which is in turn related to higher transpiration and lower WUE (Monclus et al., 2006; Pearce et al., 2005). In southern Italy (Navarro et al., 2014) *P. deltoides* genotypes “Dvina” and “Lena” were found as unsuitable SRC species in semi‐arid climatic conditions due to their low biomass production and their strong dependence on rainfall and soil water availability. In the present study, the double *P. deltoides* parentage made genotype Grimminge met these criteria and prevailed over the *P. trichocarpa* parentage (50% of the male parent). Leaves of *P. nigra* (female parentage of Oudenberg and Koster) showed a high total stomatal density (Dillen et al., 2008), similar to *P. deltoides,* but with smaller stomata. This last feature of *P. nigra* (the smaller stomatal size) might partly explain the low transpiration in genotype Koster, prevailing over the *P. deltoides* parentage component.

Poplars have been generally characterized as isohydric species (Tardieu & Simonneau, 1998), but recent studies have stated that hybrid poplars actually vary widely in their stomatal sensitivity to SWC and VPD (Arango‐Velez et al., 2011; Attia, Domec, Oren, Way, & Moshelion, 2015; Silim et al., 2009). In the present study, the genotypes Koster and Bakan featured the typical water‐conserving behavior of the isohydric species, as the values of Ψ_l_ did not change as much as those of *g*_s_ over the course of the growing season. In isohydric plants, Ψ_l_ does not vary much with changing levels of SWC or VPD, because these plants progressively close the stomata as a result of limited SWC or increased evaporative demand (e.g., maize (Tardieu, Zhang, & Gowing, 1993; Tardieu & Simonneau, 1998), tomato (Sade et al., 2009) and cowpea (Bates & Hall, 1981; Moreno, 2009)); water flow is tightly regulated by an effective stomatal control. In contrast, the behavior of genotype Grimminge was more in line with the anisohydric species, revealing a “risk‐taking” behavior, as the Ψ_l_ decreased to maintain high the *g*_s_ as SWC decreased or VPD increased over the course of the growing season. Stomatal conductance was controlled through variations on Ψ_l_, like in other anisohydric species (e.g., sunflower (Tardieu, Lafarge, & Simonneau, 1996; Tardieu & Simonneau, 1998), barley (Borel, Simonneau, This, & Tardieu, 1997) and almond trees (Wartinger, Heilmeier, Hartung, & Schulze, 1990)).

Bakan and especially Koster showed a gradual pattern of stomatal closure in response to soil drying or to higher VPD during summer months, which may protect trees from catastrophic xylem cavitation (Harvey & Van Den Driessche, 1997). This behavior was also confirmed by the slight increase in *K*_leaf_ with the increase in *E*_leaf_ in both genotypes, and might reduce the risk of xylem damage driven by excessive tension in the trees’ hydraulic system (Manzoni et al., 2013; Tyree & Sperry, 1988). The behavior of Grimminge was completely opposite, with high leaf and tree transpiration rates regardless the evaporative demand or the SWC, and a strong increase in *K*_leaf_ related to a strong increase in *E*_leaf_. The risk of keeping high *K*_leaf_ as well as high *g*_s_ values during conditions of low water availability of the soil and high VPD might induce a hydraulic failure (xylem embolism) in Grimminge. Over longer periods of drought stress, this would trigger the possible death of the tree (Attia et al., 2015). Notwithstanding this risk, the anisohydric behavior was suggested to be an agronomic benefit, as anisohydric plants may overtake the isohydric plants in terms of growth and yield (Attia et al., 2015; McDowell et al., 2008; Sade, Gebremedhin, & Moshelion, 2012). In our study, Bakan and Koster (isohydric genotypes) were more productive and tended to use water more efficiently to produce biomass than Oudenberg, and especially than Grimminge (anisohydric ones; see further below).

With regard to the daily canopy transpiration, the average daily *E*_c_ value for our site (2.1 mm/day) was within the range of 1–8 mm/day reported for poplar stands of different genotypes, stand age and geographic locations in temperate climate zones (Meiresonne et al., 1999). The daily average maximum *E*_c_ value and even the highest daily *E*_c_ value obtained by genotype Grimminge were within the range of 4.8–10.7 mm/day reported for poplar stands of different genotypes, stand age, and locations in temperate regions of Europe (Fischer et al., 2013). Similar *E*_c_ values (average and maximum of 2.2 and 6.7 mm/day, respectively) to those displayed in the present study were reported for a poplar plantation of *P. maximowiczii* × *P. nigra* using the THB method for measuring sap flow (Petzold et al., 2011). The total canopy transpiration of our study (441 mm/year on average) can only be compared with the transpiration results reported for a poplar plantation in Germany (Petzold et al., 2011) using sap flow measurements (THB method) throughout the growing season (475 mm/year), although the differences among genotypes were notable. Genotypes Bakan and Koster transpired 30% less (340 mm/year on average) and Grimminge 30% more (618 mm/year) than the genotypes in the study of Petzold et al. (2011). Other studies cannot be compared to ours as sap flow measurements were only taken over a short period of time (days or weeks; Hinckley et al., 1994; Hall et al., 1998; Allen et al., 1999) and no data of transpiration for the entire growing season are available. Our observations agree with the results of studies using sap flow + modelling (Meiresonne et al., 1999), or eddy covariance (Migliavacca et al., 2009), or the modelling + the BREB (Bowen ratio/energy balance) approach (Fischer et al., 2013) for obtaining water use data of poplar SRC in Europe. The annual water use of an SRC in the United Kingdom (Hall et al., 1996) and of a plantation for paper production in Germany (Petzold et al., 2011) was higher than that of agricultural crops and deciduous forest stands. Instead, in this study, the *E*_c_ findings for Bakan and Koster (340 mm/year) suggest a lower water use for poplar SRC cultivation with these genotypes as compared to some agriculture crops, as alfalfa (490 mm/year, Russelle et al., 2001), winter wheat and sugar beet (460 and 493 mm/year, respectively, Haferkorn, 2000). This also suggests a more competitive water use by these SRC genotypes in comparison with traditional agricultural crops, enabling SRC cultivation on land that is not assigned to food production.

In this study, for scaling *F*_s_ to *E*_c,_ we have preferred the sapwood area‐based scaling approach, for the multistem SRC plantation, rather than a scaling approach based on leaf area. In terms of measurement errors, the uncertainties associated with the upscaling of *F*_s_ to *E*_c_ with this approach could be linked to the fact that whole‐tree transpiration was estimated by summing the contributions from all stems, but stems with diameters <14 mm were not considered. If the contribution of these stems to the whole‐tree transpiration had been included, *E*_c_ could have been even higher than reported. This was not accounted as, the most stems smaller than 14 mm were leafless or if present, they were few and placed in the lower canopy layer, where the transpiration is negligible in a very dense plantation as a poplar SRC, where canopy reached the full (Heilman et al., 1996; Hinckley et al., 1994). After having fit a linear relationship between stem diameter and sap flow rate in poplars under SRC, other studies showed that the highest contribution to whole‐tree transpiration was provided by stems with diameters larger than 14 mm (Tricker et al., 2009). Another consideration for choosing the sapwood area‐based scaling of *F*_s_, was the assumption that the whole‐stem cross‐section consisted of conducting sapwood (see Figure 2), an assumption confirmed in studies with single‐stem poplars (Bloemen et al., 2017; Zhang et al., 1997) and with other single‐stem trees (Tang et al., 2006).

The higher values of WUE in Bakan and Koster should be interpreted as a low total seasonal *E*_*c*_ or the ability to produce high biomass yields with low water inputs. Differences in WUE can be identified by differences in carbon isotope ratios in trees, and genotypes with high carbon isotope discrimination (Δ ^13^C) have been usually related with higher biomass production and lower WUE (Farquhar, Ehleringer, & Hubick, 1989). However, the occurrence of a significant genotypic variability for productivity and for Δ ^13^C (used as a proxy of WUE) in poplars and other tree species has been demonstrated, including a negative relationship between biomass and Δ ^13^C (Cassasoli et al., 2004; Guehl, Nguyen‐Queyrens, Loustau, & Ferhi, 1994; Monclus et al., 2009; Zhang, Zang, & Li, 2004) or an opposite pattern (Marron et al., 2005; Rae, Robinson, Street, & Taylor, 2004). WUE in poplars is highly heritable (Monclus et al., 2005, 2006) and there is generally no trade‐off between WUE and productivity. Thus, the relationship between productivity and WUE seems to be a function of the genetic background and probably of the parentage, although further studies are needed to identify the genomic determinants of productivity and WUE via a QTL (Quantitative Trait Loci) approach. Nowadays there are not enough anchored QTLs on the poplar genome to identify and analyze the underlying large candidate gene lists (Monclus et al., 2012; Muchero et al., 2013; Rae, Street, Robinson, Harris, & Taylor, 2009) for these traits.

In conclusion, genotypes Bakan and Koster with their conservative water behavior (effective stomatal regulation) might be best suited for SRC plantations in low water input systems, with little or no irrigation. Instead, in regions such as frequently flooded marginal lands where available water is not a concern (e.g., Flanders, Belgium), genotype Grimminge might achieve a successful drainage of the flooded lands due to its water‐spending behavior (high transpiration rates). This also implies that specific genotypes might better tolerate environmental changes linked to projected climate changes effects, as for instance warming (water shortage) and flooding (water excess) conditions.
