# Supplementary material for: Genotypic variation in transpiration of coppiced poplar during the third rotation of a short‐rotation bio‐energy culture
Source: Glob Change Biol Bioenergy. 2018 Jun 4;10(8):592–607. doi: 10.1111/gcbb.12526 (PMC6109959; doi:10.1111/gcbb.12526)
Supplement: Supplementary file 1 [file GCBB-10-592-s001.docx]

**
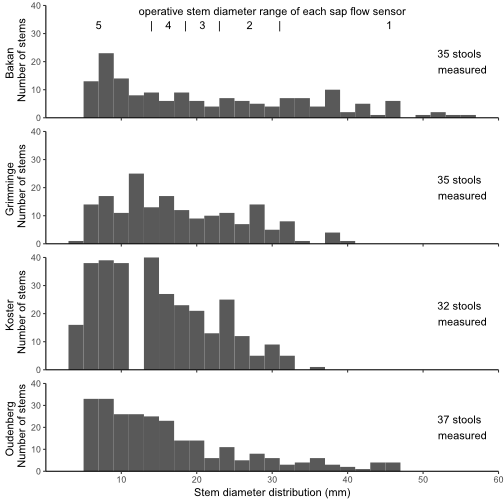
**

**Figure S1.** Number of stems of each stem diameter class (distribution in 2 cm *DHB* classes) for genotypes Bakan, Grimminge Koster and Oudenberg, after a detailed inventory made in January 2016. In the upper part of figure, the operative stem diameter range of each sap flow sensor is represented; 1 = SGB35 (>31 mm), 2 = SGEX25 (23-31 mm) 3 = SGEX19 (18.5-23 mm), 4 = SGEX16 (14-18.5 mm) and 5 = no SF sensor (<14 mm).

**Table S1.** Parameters used for scaling sap flow rate (*F*_s_) to total transpiration per unit of ground area (*E*_c_) during the 2016 growing season, according to the sapwood area-based approach. Total number of stems per tree (SN), operative stem diameter range (n) of each sap flow sensor (from 1 to 5), allometric equations; *d*=*DHB*/a where *DHB* = stem diameter at 1.30 m, *d* = stem diameter at 0.22 m and a = constant, and ground surface area per tree (*SA*) for four poplar genotypes.

| **Parameters** | | **Poplar genotype** | | | |
| --- | --- | --- | --- | --- | --- |
|  | | **Bakan** | **Grimminge** | **Koster** | **Oudenberg** |
| SN | | 4 | 5 | 10 | 7 |
| Operative stem diameter range (n) | **1**-SGB35  (>31 mm) | 1 (2)^c^ | 1 (1) | 0 | 1 (1) |
|  | **2**-SGEX25  (23-31 mm) | 1 (3) | 1 (2) | 2 (2) | 1 (2) |
|  | **3**-SGEX19  (18.5-23 mm) | 0 | 1 (2) | 1 (1) | 1 (2) |
|  | **4**-SGEX16  (14-18.5 mm) | 1 (1) | 1 (1) | 2 (3) | 1 (1) |
|  | **5**-no SF sensor^a^ (<14 mm) | 1 | 1 | 5 | 3 |
| Allometric equations | a | 0.82 | 0.80 | 0.77 | 0.86 |
|  | *r*^b^ | 0.99 | 0.98 | 0.98 | 0.98 |
| *SA* (m^2^) | | 1.49 | 1.70 | 1.53 | 1.41 |

^a^ Stems within the operative stem diameter range 5 were not taken into account when calculating whole-tree transpiration.

^b^ All regression coefficients were significant at a level of *P* < 0.001.

^c^ Numbers within the parentheses are stems monitored by sap flow sensors in that operative stem diameter range. Numbers outside the parentheses are the average stem number per stool in each operative stem diameter range. The recorded data of the stems monitored by sap flow sensors (within parentheses) were averaged and multiplied by the stem number (outside parentheses).
